# Distribution of Neuroendocrine Cells in the Transition Zone of the Prostate

**DOI:** 10.1155/2017/8541697

**Published:** 2017-03-01

**Authors:** Yuki Kyoda, Koji Ichihara, Kohei Hashimoto, Ko Kobayashi, Fumimasa Fukuta, Naoya Masumori

**Affiliations:** Department of Urology, Sapporo Medical University School of Medicine, Sapporo, Japan

## Abstract

*Objectives*. To evaluate the distribution of neuroendocrine (NE) cells which may influence the development of benign prostatic hyperplasia (BPH) in the transition zone (TZ).* Methods*. We reviewed specimens from 80 patients who underwent radical prostatectomy in our institution and evaluated the density of NE cells in the TZ. They were histologically classified into 3 groups: those with no adenomatous nodule in the TZ (group A), those with small nodules with normal epithelium and stroma around them in the TZ (group B), and those with large nodules occupying the TZ (group C). In the patients of group B, intra-adenoma (adenomatous nodules) and extra-adenoma (normal tissue) NE cells in the TZ were separately counted.* Results*. There were 22, 23, and 35 patients in groups A, B, and C, respectively. The median density of NE cells in the TZ of group B patients, 2.80/mm^2^, was significantly higher than that of NE cells in group A, 1.43/mm^2^, and group C, 0.61/mm^2^ (*p* < 0.001). In group B, the median density of extra-adenoma NE cells was significantly higher than that of intra-adenoma.* Conclusions*. Many NE cells exist around small adenoma in the TZ. NE cells may influence the initial growth of BPH in a paracrine fashion.* Trial Registration*. This study approved by our institutional review board was retrospectively registered (#272-14).

## 1. Introduction

Benign prostatic hyperplasia (BPH), which is one of the major causes of the decline in the quality of life caused by lower urinary tract symptoms, is a common histological finding in elderly men. Although prostate volume increases with age [1–3], the biological mechanism of BPH development remains unknown, though several reports have suggested the involvement of chronic inflammation and hormonal alterations [4, 5].

The prostate gland consists of a complex ductal system lined with basal and luminal cells, as well as neuroendocrine (NE) epithelial cells. Prostatic NE cells play a role in homeostasis in the prostate by producing a variety of neurosecretory products having growth-promoting activities, including serotonin, calcitonin, and parathyroid hormone-related peptides [6, 7]. Only a few studies have reported the relation between NE cells and BPH. Cockett et al. suggested that NE cells influenced the development of early nodular hyperplasia in BPH [8]. On the other hand, Manjurul Islam et al. reported there was no correlation between them [9]. Thus, the role of NE cells in the development of BPH is controversial. In this study, we evaluated the distribution of NE cells in the prostatic transition zone (TZ), where adenomatous nodules mainly appear, according to the status of the adenoma in the TZ and estimated prostate volume (PV).

## 2. Materials and Methods

### 2.1. Patients

Data on patients who underwent radical prostatectomy (RP) from January 2009 through October 2014 were extracted from medical charts and pathological specimens. Patients who received neoadjuvant androgen deprivation therapy or were administered a 5*α*-reductase inhibitor and had a tumor with a maximum diameter of 30 mm or more, or whose cancer volume was calculated to be 14 ml or more, were excluded from this study because there was a high probability that such large cancer occupied more than half of the whole prostate. Our institutional review board approved the study (#272-14).

### 2.2. Pathological Specimens

The RP specimens were fixed in 15% neutral-buffered formalin (Wako Pure Chemical Industries, Ltd., Osaka, Japan) for 48–96 h. Whole-organ prostate specimens were serially sectioned perpendicular to the rectal surface at 5-mm intervals. The specimens were embedded in paraffin, cut into 5-*μ*m sections, and stained with hematoxylin and eosin.

Immunohistochemical staining for all specimens was carried out using monoclonal antibodies for chromogranin A. It was done on 4-*μ*m-thick paraffin sections cut from the formalin-fixed tissues. Heat-induced epitope retrieval was performed in Tris-EDTA buffer (10 mM Tris Base, 1 mM EDTA solution, 0.05% Tween 20, pH 9.0). The sections were then incubated in 3% H_2_O_2_ for 10 minutes to eliminate endogenous peroxidase activity. The primary rabbit anti-chromogranin A polyclonal antibody (1 : 100, Dako, Glostrup, Denmark) and horseradish peroxidase-conjugated anti-rabbit secondary antibody were used for 60 minutes and 30 minutes at room temperature, respectively. Color development was accomplished with 3,3′-diaminobenzidine. The nuclei were then counterstained with hematoxylin. Only manifest cytoplasmic staining was defined as a positive reaction. Negative controls were incubated with normal rabbit serum instead of the polyclonal antibody.

### 2.3. Evaluation of NE Cells

**Figure 1:**
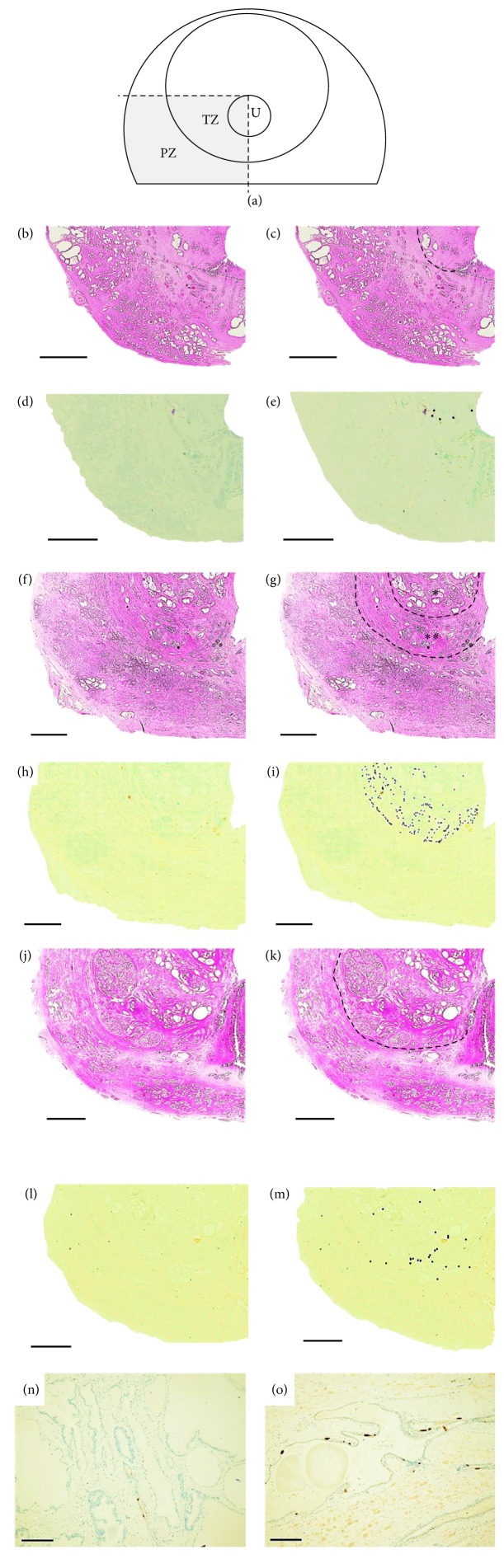
(a) NE cells in the transition zone of a dorsal section obtained from a radical prostatectomy specimen were evaluated. TZ: transition zone, PZ: peripheral zone, U: lumen of urethra. (b–e) Group A. (f–i) Group B. (j–m) Group C. (b), (f), and (j) Gross anatomy and histology of a dorsal prostate section containing verumontanum (hematoxylin and eosin staining). (c), (g), and (k) Transition zone. (g) In group B, the TZ included 2 areas, intra-adenoma and extra-adenoma, which are divided by a dotted line. ^*∗*^Intra-adenoma, ^*∗∗*^extra-adenoma. (d), (h) and (l) Immunoreactivity against chromogranin A. (e), (i), and (m) Chromogranin A-positive cells in the transition zone were manually dotted. (n) High magnification of chromogranin A-positive NE cells in the intra-adenoma area of a group B patient. (o) High magnification of chromogranin A-positive NE cells in the extra-adenoma area of a group B patient. Scale bars: (b–m), 4 mm; (n) and (o), 200 *μ*m.

We evaluated the distribution of NE cells in the TZ by using a dorsal section containing verumontanum tissue because Manjurul Islam reported that many NE cells appear in it [9] and McNeal reported that this level of section contains TZ [10] (Figure 1(a)). No adenocarcinoma existed in this section. We histologically divided the prostates into 3 groups with the focus on benign hyperplastic adenomatous nodules of BPH, with group A having no nodule in the TZ (Figures 1(b) and 1(c)), group B having small nodules with normal epithelium and stroma around them in the TZ (Figures 1(f) and 1(g)), and group C having a TZ occupied by large nodules (Figures 1(j) and 1(k)). McNeal reported that BPH evolved through three processes: early diffuse gland growth, small nodule proliferation, and later nodule enlargement [10]. In group B, we defined 2 areas in the TZ of the prostate: intra-adenoma, which was the area composed of clusters of adenomatous nodules, and extra-adenoma, which was the area composed of normal tissue outside the intra-adenoma area in the TZ (Figure 1(g)). Figures 1(d), 1(h), and 1(l) show the gross anatomy and histology of the immunohistochemical staining using chromogranin A in serial hematoxylin and eosin-stained sections (Figures 1(n) and 1(o)). Furthermore, we used pictures with manually applied dots to indicate chromogranin A positive NE cells in the TZ (Figures 1(e), 1(i), and 1(m)) because the magnification in Figures 1(d), 1(h), and 1(l) was too low to easily identify NE cells.

We calculated the density of NE cells in the TZ. Stained cells were manually counted in high power fields of a microscope (BZ-9000, KEYENCE, Tokyo, Japan) by a single urologist and the intra-adenoma and extra-adenoma areas and the whole TZ were automatically calculated using software (BZ-X analysis application, KEYENCE, Tokyo, Japan).

In addition, we evaluated the density of NE cells based on the estimated PV and TZ volume (TZV) calculated by transrectal ultrasound (TRUS-PV and TRUS-TZV, resp.). TRUS was performed with a 7 MHz biplane probe by urologists using the same equipment (Flex FOCUS 400, BK Medical) in the left lateral decubitus position. The maximum transverse and longitudinal diameters of the whole prostate and the TZ were measured, and PV and TZV were calculated by the following formula: (*π*/6) × width × height × length. We did not measure the TZV of the prostates in group A by TRUS because it was difficult to judge the boundary between the TZ and peripheral zone (PZ) due to the lack of adenoma [11].

### 2.4. Statistical Analysis

Statistical comparisons were made using the nonparametric Kruskal–Wallis test and Mann-Whitney *U*-test. *p* < 0.05 was considered to be statistically significant. These analyses were performed using SPSS®, v. 23.0 (SPSS Inc., Chicago, IL).

## 3. Results

### 3.1. Patient Characteristics

Of the 80 patients, 22 had no adenomatous nodule in the prostatic TZ (group A), with a median PV of 23.1 ml (Table 1). Of the remaining 58 patients, who had histological adenomatous nodules in the TZ, 23 had a cluster of small adenomatous nodules in the TZ surrounded by normal tissue (group B), and in 35 the TZ was occupied by large adenomatous nodules without normal tissue (group C). Their median estimated PVs were 29.4 and 48.6 ml, respectively (*p* < 0.001). Patients in group C were significantly older than those in the other 2 groups. The maximum cancer diameter in group A was significantly larger than in the other 2 groups.

### 3.2. The Relation between NE Cell and Total PV

**Figure 2:**
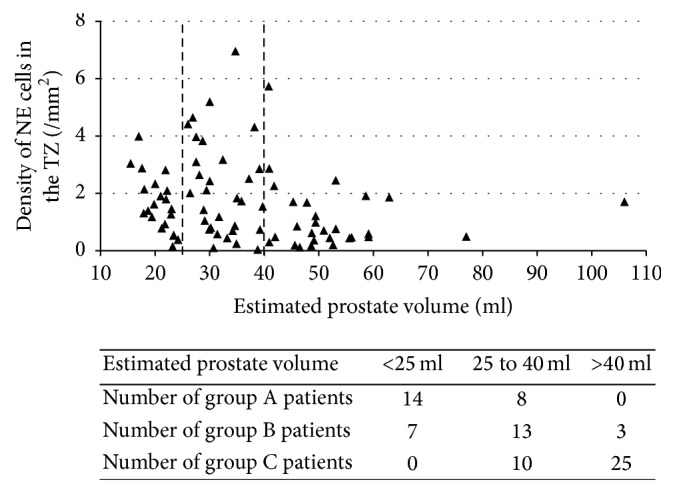
Triangle plot of density of NE cells in the transition zone based on prostate volume estimated by transrectal ultrasound in all patients. Dotted lines are drawn for estimated prostate volumes of 25 ml and 40 ml.

Figure 2 shows the density of NE cells in the TZ based on TRUS-PV. We divided the prostates into 3 groups whose estimated PVs were less than 25 ml, 25 to 40 ml, and more than 40 ml. Since Japanese men who are older than 55 years old in the general population have a median prostate volume of 23.9 ml [2], 25 to 40 ml seems to be slightly enlarged. In addition, approximately 10% had an obviously enlarged prostate with a PV of more than 40 ml, in this community-based study. Thus, the cutoffs 25 and 40 ml had clinical meaning in the current study. In group A, a PV of less than 25 ml was most common (14 of 21 patients). On the other hand, in groups B and C, the most frequently observed prostate volumes were 25 to 40 ml (13 of 31) and more than 40 ml (25 of 28), respectively. A scattergram showed that patients having PVs of 25–40 ml had a higher density of NE cells, with a median of 1.82/mm^2^ (IQR: 0.72–3.16), than the groups smaller than 25 ml, 1.45/mm^2^ (IQR: 0.84–2.23), and greater than 40 ml, 0.65/mm^2^ (IQR: 0.43–1.70) (*p* = 0.013, Kruskal–Wallis test).

### 3.3. The Relation between NE Cell and Adenomatous Nodule

**Figure 3:**
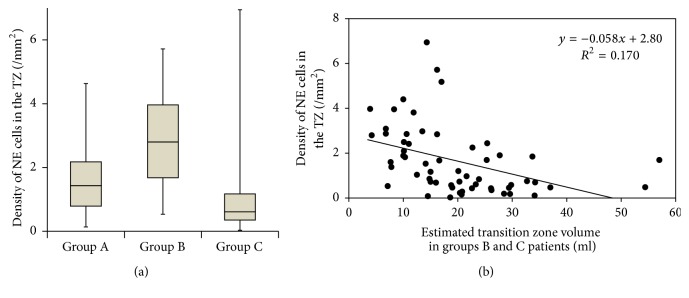
(a) Bar graph plot of density of NE cells in the prostatic transition zone for patients with no adenoma (group A), small adenoma (group B), and large adenoma (group C). Horizontal lines within boxes, boxes, and bars represent the median, interquartile range, and range, respectively. *p* < 0.001 (Kruskal-Wallis test). (b) Scatter plot of density of NE cells for groups B and C in the transition zone based on the transition zone volume estimated by transrectal ultrasound. They have an inverse correlation. We did not measure the TZV in group A by TRUS because it was difficult to judge the TZ due to the lack of adenoma.

Group B had a higher density of NE cells than the other 2 groups in the TZ of prostate. In group A the median density was 1.43/mm^2^ (IQR: 0.78–2.18); in group B it was 2.80/mm^2^ (IQR: 1.68–3.96); in group C it was 0.61/mm^2^ (IQR: 0.35–1.17), (*p* < 0.001, Kruskal–Wallis test, Figure 3(a)).  Figure 3(b) shows a scatter plot of the density of NE cells in the TZs of the prostates in groups B and C based on TRUS-TZV. This graph suggested an inverse correlation between TRUS-TZV and the density of NE cells in the prostatic TZ.

**Figure 4:**
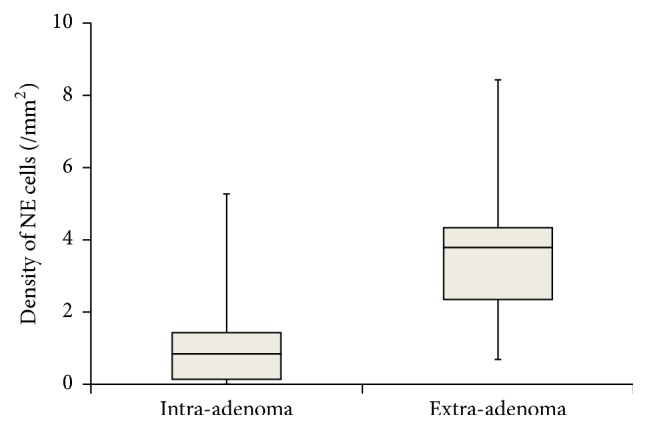
Bar graph plot of density of NE cells for intra-adenoma and extra-adenoma in prostatic transition zone. Horizontal lines within boxes, boxes, and bars represent the median, interquartile range, and range, respectively. *p* < 0.001 (Mann–Whitney *U* test).

In the 23 patients with small adenomatous nodules (group B), the density of extra-adenoma NE cells was higher than that of intra-adenoma NE cells (median 3.79/mm^2^ (IQR: 2.35–4.33) versus 0.85/mm^2^ (IQR: 0.14–1.43), *p* < 0.001, Mann–Whitney *U* test, Figure 4). The density of extra-adenoma NE cells in group B was higher than that in the TZ of group A when focusing on the normal tissue (*p* < 0.001, Mann–Whitney *U* test). On the other hand, the density of intra-adenoma NE cells in group B was comparable to that in the TZ of group C when focusing on the adenomatous nodules (*p* = 0.843, Mann–Whitney *U* test).

## 4. Discussion

Several studies have proved that NE cells have an impact on prostate development. The appearance of NE cells in the prostate in the early fetal period and their absence in less differentiated glands possibly indicates that differentiation of NE cells is associated with glandular maturation in the early period [12]. Cheng et al. reported that prostate hypotrophy was induced in transgenic mice in which the number of NE cells in the prostate was substantially decreased. Thus NE cells play an important role in prostate development [13]. NE cells secrete many peptides, for example, vascular endothelial growth factor and transforming growth factor-*α*, that seem to affect the prostatic development [14].

In this study, we demonstrated a correlation between NE cells and the initial development of BPH since the prostate with small adenomatous nodules, which were presumed to be the early stage of BPH, tended to contain a high density of NE cells in the TZ. In addition, increased density of NE cells was observed only in extra-adenoma in the prostate with small nodules. It was shown that there was a strong relation between the density of NE cells in the TZ and TRUS-TZV in groups B and C. These results may indicate that the paracrine effect of NE cells existing in the normal glands located in the periphery of the TZ influences the growth of the early stage of adenoma in the TZ. Cockett et al. previously reported a high volume of NE cells in and around small adenoma, although they did not quantify the NE cells [8]. To the best of our knowledge, our study is the first report quantifying them. Furthermore, the 80 samples are twice as many as in the study reported by Cockett et al., as prostatectomy specimens can be easily obtained because of the global increase of the operation volume for prostate cancer recently [15]. In addition, this is the first attempt to focus on the NE cells only in the prostatic TZ. Since adenomatous nodules, which are the main pathological finding of BPH, mostly arise in the TZ [16], NE cells in the TZ should be evaluated to determine the paracrine effect [17]. NE cells in the PZ are unlikely to affect the development of BPH. In previous studies, the investigators often counted the NE cells as a proportion of total epithelial cells [12, 18, 19] but we evaluated them by the density in all cells in the TZ because we considered that NE cells had an effect on not only epithelial cells but also stromal cells in a paracrine fashion [17]. This is the first attempt to do so.

Chronic inflammation seems to influence the later period of BPH because it is more frequently observed in the large prostate [20]. On the other hand, the factors affecting the early stage of BPH remain unclear. We assume that NE cells have an effect in this period and perhaps from childhood because Xue et al. reported that there were individual differences in the density of prostatic NE cells in the prenatal period [12].

A conclusion has not been reached concerning the reason why the density of NE cells is reduced in the late stage of BPH. We propose two speculations. First, the number of NE cells may decrease as a result of finishing the activated period due to aging or negative feedback from adenomatous nodules. If group A patients who had small prostates were to be divided into two patterns, growing or being atrophic, the density of NE cells in the TZ of the growing prostate might be high, whereas that of the atrophic prostate might be low. Second, the extra-adenoma NE cells might disappear by being compressed between the grown adenomatous nodules and the PZ. In group C, the extra-adenoma area could not be microscopically identified due to the large adenomatous nodules in the TZ. Although the mechanism remains unknown, the role of NE cells in growth in large adenoma was ended and other factors might influence the further growth of large adenoma.

There are some limitations to this study. All subjects in this study were diagnosed with prostate cancer. A relationship between NE cells and cancer cells might exist regardless of the exclusion criteria about cancer size and the absence of cancer cells in the evaluated sections. Prostate cancer cells might have an impact on benign prostatic tissue since more than 30% of patients with prostate cancer have NE cells in the prostate cancer cells [21–23]. Another limitation is that we evaluated the distribution of the NE cells using only one section. Thus, it may not be representative for the whole TZ. However we selected a slide with the TZ at the same level of section containing verumontanum tissue [9, 10]. In addition, we did not count the NE cells in the PZ because it was located far from the adenomatous nodules. Furthermore, NE cells were evaluated only by chromogranin A. However, there is no marker that is positive in all NE cells. It is reported that the positive rates of chromogranin A or serotonin in NE cells are higher than those of any other markers [24, 25].

## 5. Conclusions

The density of NE cells around small adenoma in the TZ of the prostate was high. We propose that NE cells have an effect on the growth of adenoma in the early stage of BPH in a paracrine fashion. However, further study of the relationship between NE cells and BPH is needed.

## Figures and Tables

**Table 1:** Baseline characteristics of the 3 groups of patients with prostate cancer.

Characteristics/group^a^	A	B	C	*p* value
Patients (*n*)	22	23	35	
Age (years)				0.044^b^
Median	64	63	68	
Range	54–75	50–74	54–75	
PSA (ng/ml)				0.205^b^
Median	5.9	5.7	6.8	
Range	3.5–19.8	4.1–10.7	4.1–21.8	
Maximum cancer diameter (mm)				0.016^b^
Median	16	12	11	
Range	2–28	2–24	3–30	
Pathological Gleason sum [*n* (%)]				0.299^c^
6	1 (4.5)	2 (8.7)	7 (20.0)	
7	12 (54.5)	16 (69.6)	22 (62.9)	
8	8 (36.3)	3 (13.0)	2 (5.7)	
9	5 (22.7)	2 (8.7)	4 (11.4)	
Pathological stage (%)				0.938^c^
pT2a	11 (50.0)	14 (60.9)	23 (65.7)	
pT2b	3 (13.6)	2 (8.7)	3 (8.6)	
pT2c	6 (27.3)	6 (26.1)	7 (20.0)	
pT3a	1 (4.5)	1 (4.3)	1 (2.9)	
pT3b	1 (4.5)	0 (0)	1 (2.9)	
Estimated prostate volume (ml)				<0.001^b^
Median	23.1	29.4	48.6	
Range	15.5–35.8	17.0–47.8	29.1–106.0	
Estimated transition zone volume (ml)				<0.001^b^
Median	—	10.1	23.3	
Range	—	3.9–17.0	12.5–57.0	

^a^Group A had no adenomatous nodule, group B had small nodules, and group C had large nodules in the prostatic transition zone in pathological findings.

^b^Kruskal-Wallis test.

^c^Chi-square test.
